# Deep neuromuscular blockade during radiofrequency catheter ablation under general anesthesia reduces the prevalence of atrial fibrillation recurrence when compared to moderate neuromuscular blockade: A randomized controlled trial

**DOI:** 10.1371/journal.pone.0302952

**Published:** 2025-01-21

**Authors:** Eun Jung Oh, Jae-Geum Shim, Suyong Jeon, Eun Ah Cho, Sung Hyun Lee, Taeho Jeong, Jin Hee Ahn

**Affiliations:** Department of Anesthesiology and Pain Medicine, Kangbuk Samsung Medical Center, Sungkyunkwan University School of Medicine, Seoul, Republic of Korea; Scuola Superiore Sant'Anna, ITALY

## Abstract

**Background:**

Proper anesthesia management is required to maintain immobilization and stable breathing of the patient to improve catheter contact and stability during catheter ablation for PVI. However, it remains unclear whether the depth of neuromuscular blockade affects the results of RFCA under general anesthesia.

**Methods:**

The patients were randomly assigned to either the moderate neuromuscular blockade group (Group M, train-of-four 1 to 2) or the deep neuromuscular blockade group (Group D, posttetanic count 1–2). The primary outcome was the 12-month AF recurrence rate using confirmed electrocardiographic diagnosis after the ablation procedure at two different neuromuscular blockade depths.

**Results:**

Total 94 patients (47 in each group) were included in the analysis. Recurrence of AF during the A 12-month follow-up was 12 (25%) in group D and 22 (46%) in group M. The AF recurrence rate was significantly higher in group M (p =  0.03). The relative risk (RR) for the risk of 12-month AF recurrence was 0.545 in group D. AF symptom recurrence was observed during the A 12-month follow-up in 12 (25%) and 26 (54%) patients in groups D and M, respectively.

**Conclusions:**

Compared to moderate neuromuscular blockade, deep neuromuscular blockade while performing RFCA under general anesthesia reduced 12-month AF recurrence rate. Deep neuromuscular blockade under general anesthesia is thought to increase the success rate by providing a stable surgical environment during the RFCA procedure.

**Trial registration:**

Clinical trials of Korea KCT 0003371

## Introduction

Radiofrequency catheter ablation (RFCA) is a widely used therapy for atrial fibrillation (AF) [1]. Despite its widespread use, the AF recurrence rate following a single RFCA procedure can reach up to 49% [2,3]. Steps for the RFCA procedure include catheter cannulation, arrhythmia induction, and pulmonary vein isolation (PVI), and these cause pain and chest discomfort. Therefore, proper anesthesia management is required to maintain immobilization and stable breathing of the patient to improve catheter contact and stability during catheter ablation for PVI. Appropriate anesthetic management may have an impact on the success of RFCA. In a previous study, when RFCA was performed under general anesthesia, the fluoroscopy and procedure times were shorter when compared RFCA performed under sedation, and the recurrence rate was lesser (12% in general anesthesia and 31% in sedation) [4,5]. However, anesthetics have an antiarrhythmic effect [6–8]. The success of ablation through arrhythmia induction is determined at the end of the procedure, and there is a possibility that the remnant connection may be masked. In addition, the depth of anesthesia can also affect recurrence of arrhythmia. The arrhythmia recurrence rate according to the bispectral index (BIS) level during general anesthesia was 51.7% vs. 15.3%, and the recurrence rate was significantly higher in the low BIS group (<40) compared to the high BIS group (>50) [6,9]. It is known that the depth of anesthesia indicated by the BIS value affects unresponsiveness during induction of ventricular tachycardia, which is the final step of RFCA to confirm residual arrhythmias [6,9].

Previous studies have shown that the outcome of RFCA is influenced by the type and depth of anesthesia [6,9]. However, it remains unclear whether the depth of neuromuscular blockade affects the results of RFCA under general anesthesia. Therefore, we hypothesized that the depth of neuromuscular blockade during anesthesia would affect the AF recurrence rate after RFCA.

## Materials and methods

### Study population

This study selected patients based on the American Society of Anesthesiologists physical status classification (ASA PS) I to IV. Patients aged 20–70 years who were scheduled to undergo RFCA for AF at Samsung Medical Center between February 2019 and May 2019 were selected and followed up for 1 year. The clinical trial was registered prior to patient enrollment, along with the registry (CRIS, https://cris.nih.go.kr), registration number (KCT 0003371, November 23, 2018), principal investigator’s name (JSJ), and date of registration. All patients who received general anesthesia for RFCA and those who did not undergo RFCA before were included in the study. Exclusion criteria included age <19 years, previous history of RFCA, expected aspiration pneumonia, unstable vital signs, renal disease, hepatic disease, metabolic disease, emergent procedure, and pregnancy. This study was approved by the Institutional Review Board of Samsung Medical Center, and all study protocols were carried out in accordance with the Samsung Medical Center Institutional Review Board guidelines and regulations. All patients provided written informed consent prior to the procedure (Institution Review Board Number: SMC 2018-08-159, November 02, 2018).

#### Randomization and blinding.

The patients were randomly assigned to either the moderate neuromuscular blockade group (Group M) or the deep neuromuscular blockade group (Group D). Randomization was performed using a random number list (http://www.randomizer.org). Sequential numbers were concealed in opaque envelopes and opened by one of the two anesthesiologists (JHA and JEY) just before anesthesia induction. The cardiologist performing the RFCA procedure was blinded to the assigned group. The intraoperative recording of study data was carried out by a nurse who was not involved in the study, and the data were coded and managed by an anesthesiologist who did not participate in this study. After the 12-month follow-up period, the results of AF recurrence, symptom recurrence, and other outcomes were obtained through electronic medical records by another anesthesiologist who did not participate in the study.

#### Anesthetic management.

Anesthesia protocol applied the standard general anesthesia guidelines of our institution except for the dose of neuromuscular blocking agent. Premedication was not administered. Before the induction of general anesthesia, standard monitoring, including electrocardiography, pulse oximetry, non-invasive blood pressure, BIS, and train of four (TOF), were performed. In addition, 2 mg/kg of 1% propofol was administrated intravenously. After confirmation of loss of consciousness, TOF was calibrated and measured every 5 min. Simultaneously, 8% sevoflurane and 0.4 mg/kg rocuronium were administrated. After 90 s, airway control was achieved using the supraglottic airway (I-gel; Intersurgical, Wokingham, UK). Anesthesia was maintained using sevoflurane (1MAC) and adjusted to maintain BIS between 40 and 60. For mechanical ventilation, the fresh gas flow was set to 3l/min, and the tidal volume was set to 8 mL/kg. If the airway pressure exceeded 20 mm Hg, the supraglottic airway was repositioned. The degree of neuromuscular blockade was evaluated using TOF-Watch SX (Organon Ltd., Dublin, Ireland), Every 15 s, the ulnar nerve was stimulated through surface electrodes with TOF mode. When the TOF count was 1 or 2, continuous infusion of rocuronium was initiated.

In group M, continuous infusion of rocuronium was administered at an initial dose of 5 mcg/kg/min and increased by 1 mcg/kg/min when the TOF count was 3 or 4, and decreased by 1 mcg/kg/min when the TOF count was 0. In group D, continuous infusion of rocuronium was administered at a dose of 8 mcg/kg/min and increased by 1 mcg/kg/min when the TOF count was 1 or 2. When the TOF count was 0, the post-tetanic count (PTC) was determined, and the rocuronium dose was adjusted to maintain PTC less than 2. When the procedure was completed, 4 mg/kg of sugammadex was administered to reverse the neuromuscular blockade in both groups.

#### Catheter ablation (PVI).

Using fluoroscopic guidance, 6-F quadripolar and 7-F duodecapolar electrode catheters were placed in the right ventricular apex, crista terminalis of the right atrium and coronary sinus via the left femoral vein. Two SL1 sheaths (St. Jude Medical, St. Paul, MN, USA) were then inserted into the LA via transseptal puncture. Heparin (Initial dose: 100 U/kg) was administered for systemic anticoagulation, and activated clotting time was checked every 30 min with a target level of 300–350 s. LA geometry was mapped using the Carto 3 system (Biosense Webster, Diamond Bar, CA, USA), and the resulting image was integrated with computed tomography images. A 3.5 mm tip contact-force (CF)-sensing irrigated tip ablation catheter (Thermocool Smartouch, Biosense Webster Inc., Diamond bar, CA, USA) was inserted into the pulmonary vein (PV), after which the operator checked the PV potential. The targeted contact force ranged from 10 to 40 g. Energy delivery was controlled with a pre-set power of 30 watts, reduced to 25 watts for the posterior wall. Ablation tags were automatically generated using the VisiTag module. The predefined parameters included catheter stability, with a motion range of ≤2 mm for more than 4 seconds, and a minimum force of ≥10 g for over 70% of the time. If these criteria were not met, tags were manually annotated by the operator when the RF duration reached 20 seconds (or 15 seconds for the posterior wall) Radio-frequency energy was applied to remove the residual potentials from the four PV antra. If the residual potential around the PV antra was ablated, but the sinus rhythm was not restored, the mitral isthmus, mitral annulus, roofline, septal line, and anterior portion of the superior vena cava were ablated. After the ablation, we confirmed the entrance block of all four pulmonary veins (PVs) and the bidirectional block of the linear lines. Successful AF ablation was confirmed through the infusion of high-dose isoproterenol and rapid atrial pacing [10].

#### Data acquisition.

Patient characteristics, including sex, age, height, weight, ASA class, underlying disease, previously prescribed drugs, history of alcohol consumption, and smoking were checked before induction of anesthesia. Hemodynamic parameters such as heart rate, blood pressure, BIS, body temperature, and TOF counts were also checked as baseline data before the induction of anesthesia. These values were recorded in chronological order: immediately after induction of anesthesia, at the beginning of the RFCA procedure, at the time of ablation in the pulmonary veins or other sites, and immediately after the procedure was completed. The operator satisfaction score was collected from cardiologist after the procedure was completed. A score of 0 was assigned when the physician was extremely dissatisfied, 1 when dissatisfied, 2 when neutral, 3 when satisfied, and 4 when extremely satisfied [11]. Any adverse events during the procedure and self-respiration recovery during pulmonary vein ablation were recorded. For all patients under general anesthesia and mechanical ventilation, regular capnography patterns were recorded. Self-respiration was recognized based on abrupt irregular capnography. Follow-up was performed 3, 6, 9, and 12 months after the procedure. In the case of undesirable symptoms, the follow-up schedule was started earlier. At each time point, 24-h Holter monitoring and echocardiography were performed to evaluate RFCA success. A cardiologist prescribed anticoagulants and antiarrhythmics according to the 2017 European society cardiology guidelines on atrial fibrillation, and anticoagulants were prescribed for the first 3 months in all patients and discontinued if there was no evidence of recurrence after the procedure [12]. Additional medications were prescribed based on the risk of thromboembolism, the presence of AF, previous stroke history, and CHA2DS2-VASc score [13].

The primary outcome was the 12-month AF recurrence rate using confirmed electrocardiographic diagnosis after the ablation procedure at two different neuromuscular blockade depths under general anesthesia.

### Statistical analysis

Based on our pilot study conducted between September 2017 and February 2018, a retrospective analysis was performed to calculate the required sample size. The analysis indicated that the 1-year recurrence rate of atrial fibrillation was 4% (1 out of 25 patients) in the deep neuromuscular blockade group and 25% (5 out of 20 patients) in the moderate blockade group. The difference in proportions between these two groups was assessed using a Z test and confirmed with a chi-square test, with a significance level (α) set at 0.05 and a desired statistical power of 0.8. Assuming a dropout rate of 10%, 48 patients per group were determined to be necessary to achieve sufficient power. Data are presented as mean ( ± standard deviation), median (interquartile range), numbers (n), and percentages (%). Continuous variables were compared using the t-test or Mann–Whitney U test, as appropriate, and the Shapiro–Wilk test was used to explore normality. Categorical variables were compared using Pearson’s chi-square or Fisher’s exact tests. The Kaplan–Meier survival curve was used to analyze time-to-event data and compare the two neuromuscular blockade depths using the log-rank test. All statistical analyses were performed using MedCalc^®^ Statistical Software version 20.014 (MedCalc Software Ltd, Ostend, Belgium; https://www.medcalc.org; 2021) and R, version 4.1.2 (The R Foundation). We used 2-tailed tests in all analyses, with P values <0.05 considered statistically significant.

## Results

**Fig 1:**
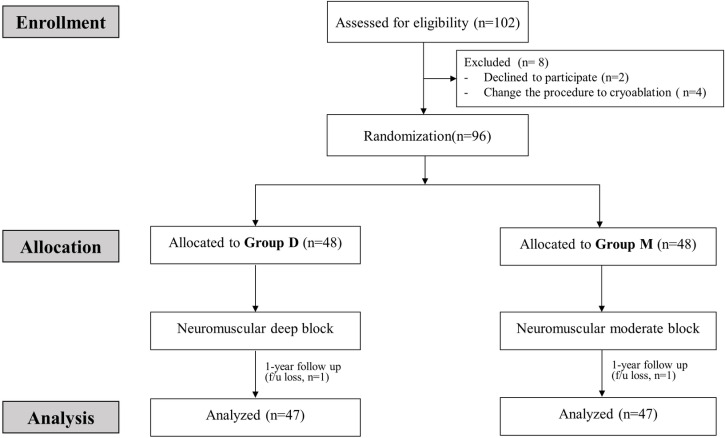
The CONSORT flow diagram.

The total study period, including a 1-year follow-up, was conducted from February 2019 to June 2020. A total of 102 patients were assessed for eligibility. Of these, six patients were excluded because they refused to participate in the study (n =  2) or the surgical plan was changed to cryoablation instead of RFCA (n =  4). Therefore, 96 patients were enrolled and assigned to either group D (n = 48) of M (n = 48). During the 12-month follow-up period, one patient in each group was lost to follow-up. Finally, data from 94 patients (47 in each group) were included in the analysis (Fig 1). Patient characteristics are shown in Table 1. There were no significant differences between the two groups. Intraoperative data are described in Table 2. The durations of anesthesia, procedure, and ablation were not significantly different between the groups. The median (interquartile range [IQR]) continuous infusion rate of rocuronium was 8 [7–10] mcg/kg/min in group D and 5 [4–5] mcg/kg/min in group M (p <  0.0001), and the median (IQR) total dose of rocuronium was significantly higher in group D (105 [87–120] mg) compared to group M (69 [55–80] mg) p <  0.0001). The number of patients who recovered spontaneous breathing during ablation was 7 (15%) in group D and 14 (30%) in group M (p =  0.085). The operator satisfaction scores tended to be higher in group D (p =  0.034).

**Table 1:** Patient characteristics.

	Group D (n = 47)	Group M (n = 47)
Sex (Female/Male)	4/43	10/38
Age, months	57 ± 9	57 ± 10
Weight, kg	72 ± 8	69 ± 9
Height, cm	169 ± 7	169 ± 8
ASA PS (I/II/III)	0/40/8	0/40/8
Arterial hypertension, n (%)	24(50)	23(48)
Diabetes mellitus, n (%)	9 (19)	5(10)
Medication use		
Current number of Antiarrhythmic drugs	2.2 ± 0.7	2.1 ± 0.6
Anticoagulants use, n (%)	42(88)	46(96)
Wafarin, n	0	1
Aspirin, n	1	1
NOAC, n (Apixaban/Edoxaban/ Rivaroxaban/ Dabigatran)	41 (7/24/8/2)	44 (12/21/10/1)
Paroxysmal atrial fibrillation, n (%)	38(79)	35(73)
Structurally normal heart, n (%)	37(77)	38(79)
Normal ejection fraction, n (%)	43(90)	40(83)
First ablation procedure, n (%)	39(81)	37(77)
Postoperative anti-arrhythmic drug, n		
Amiodarone, n	8	6
Dronedarone, n	17	16
Propafenone, n	9	14
Flecainide, n	10	11
Sotalol, n	4	1

Data are presented as the median [IQR], mean ±  SD or number (percentage).

Abbreviation: ASA PS, American society of anaesthesiologists physical status; NOAC, new oral anticoaculants.

**Table 2:** Intraoperative data.

	Group D (n = 47)	Group M (n = 47)	*P-value*
Rocuronium induction dose (mg)	30 [27, 35]	30 [25, 30]	0.089
Rocuronium continuous infusion rate (mcg/kg/min)	8 [7, 10]	5 [4, 5]	<0.0001
Total dose (mg)	105 [87, 120]	69 [55, 80]	<0.0001
Anesthesia duration (min)	157 [132, 183]	153 [130, 176]	0.555
Procedure duration (min)	135 [110, 160]	130 [110, 156]	0.543
Ablation duration (min)	81 [64, 101]	74 [59, 93]	0.100
Maximum esophageal temperature during ablation, up to ‘C	38.1 ± 0.9	37.9 ± 1.1	0.337
Self respiration recovery, n (%)	7 (15)	14 (30)	0.085
Operator satisfaction score			0.034^a^
0	0	3	
1	5	4	
2	14	16	
3	14	21	
4	13	2	

Data are presented as the median [IQR], mean ±  SD or number (percentage).

^a^P-value derived by chi-squared test for trend.

### Postoperative outcomes

**Fig 2:**
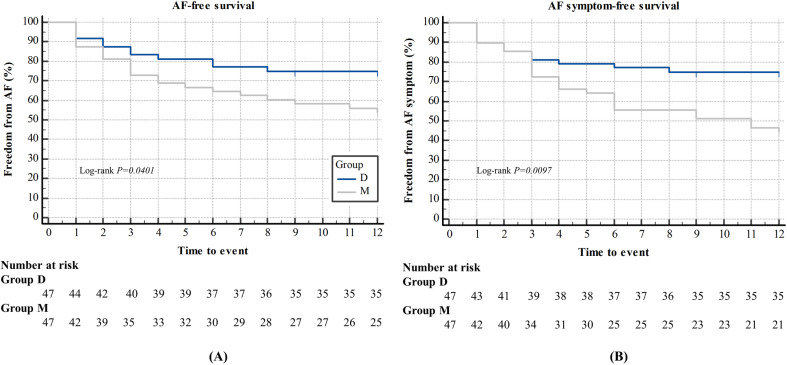
Kaplan–Meier analysis. Kaplan-Meier curves shows cumulative probability of AF-free survival (A) and AF symptom-free survival (B) according to degree of neuromuscular blockade after radiofrequency ablation procedure.

The primary outcomes are described in Table 3. Recurrence of AF during the A 12-month follow-up was 12 (25%) in group D and 22 (46%) in group M. The AF recurrence rate was significantly higher in group M (p =  0.033). The relative risk (RR) and 95% confidence interval (CI) for the risk of 12-month AF recurrence was 0.545 (0.307–0.970).Furthermore, AF symptom recurrence was observed during the A 12-month follow-up in 12 (25%) and 26 (54%) patients in groups D and M, respectively. The recurrence rate of AF symptoms was also significantly higher in group M (p =  0.003). The RR (95% CI) of AF symptom recurrence during the 12-month follow-up was 0.462 (0.266–0.801). Kaplan–Meier curves are shown in Fig 2. The cumulative probability of AF-free survival (A) and AF symptom-free survival (B) showed significant differences in relation to the depth of the neuromuscular blockade (log-rank test; p =  0.0401 and p =  0.0097). During the postoperative follow-up period, seven (15%) and three (6%) patients underwent redo-RFCA in groups D and M, respectively (p =  0.183). Furthermore, one (2%) and two (4%) patients underwent additional transthoracoscopic ablation in groups D and M, respectively (p =  1.000).

**Table 3:** Postoperative outcomes.

	Group D (n = 47)	Group M (n = 47)	*P-value*
**Recurred AF**			
Patients, n (%)	12 (26)	22 (47)	0.033
Relative risk (95%CI)	0.545 (0.307–0.970)	–	–
**Recurred Symptom**			
Patients, n (%)	12(26)	26 (55)	0.003
Relative risk (95%CI)	0.462 (0.266–0.801)	–	–
**Redo RFCA**	7 (15)	3 (6)	0.183
**TTA**	1 (2)	2 (4)	1.000

Data are presented as number (percentage) and relative risk (95% CI).

The p-value was calculated using the chi-square test or Fisher’s exact test. Abbreviation: TTA, Transthoracoscopic ablation.

## Discussion

The results of this prospective study showed that deep neuromuscular blockade could reduce the recurrence rate of AF after RFCA under general anesthesia. Compared to group M, the spontaneous self-respiration recovery incidence was lower and the operator satisfaction score was higher in group D. It is concluded that the depth of neuromuscular block during general anesthesia provides stability during the pulmonary vein isolation procedure. To the best of our knowledge, this is the first study to demonstrate that deep neuromuscular blockade during RFCA with general anesthesia is superior to moderate blockade in terms of a 1-year AF-free survival rate. Our results can serve as a guide for appropriate anesthetic management during RFCA procedures.

Pulmonary vein reconnection has a strong association with the recurrence of AF after RFCA [14,15]. Accurate and appropriate catheter contact during pulmonary vein isolation reduces the recurrence of pulmonary reconnection [16]. Compared to conscious sedation, pulmonary vein reconnection is reported to be less frequent under general anesthesia [4,5]. Neuromuscular blockade agents are key drugs for general anesthesia, they cause paralysis and prevent abrupt movements of the patient, thus enabling mechanical ventilation and providing stable procedure environment [4,17]. Periodic thoracic movements using mechanical ventilation can predict changes in catheter placement over the ventilation cycles. In addition, minimizing respiratory movement using high-frequency jet ventilation can be an effective option to improve catheter stability. Bhradeev et al. demonstrated that the high-frequency jet ventilation group showed a lower 1-year paroxysmal AF recurrence rate compared to the group without high-frequency jet ventilation (27.3% vs. 47.3%) [18].

Phrenic nerve stimulation and abrupt diaphragmatic contraction may occur during pulmonary vein isolation. Unexpected diaphragm contraction is one of the reasons why operators adjust catheter placement and perform re-mapping. In our study, although not statistically significant, the frequency of recovery of spontaneous breathing due to phrenic nerve stimulation during the procedure was lower in the deep neuromuscular blockade group (15% versus 30%). Abrupt movement of the patient may occur when the mechanical ventilation cycle and the patient’s respiratory cycle are not synchronized. Depending on the depth of neuromuscular blockade, unexpected diaphragmatic contraction can be prevented. The diaphragm is more resistant to neuromuscular blockade agents than other peripheral muscles, such as the abductor pollicis, where TOF is usually monitored. It is known that diaphragm movement can be inhibited in PTC counts < 12–15 [19]. However, in our study, diaphragm movement was observed in 15% of group D patients despite maintaining PTC <2. Direct nerve stimulation through the catheter cannot be completely inhibited, but, the frequency of diaphragm contraction can be decreased using deep neuromuscular blockade. In addition, the tendency for the operator satisfaction score to be higher in group D means that the occurrence of abrupt movement can be reduced, contributing to stability during the procedure, thereby increasing the RFCA success rate.

Compared to the moderate neuromuscular blockade group, the deep neuromuscular blockade group showed higher 1-year symptom-free survival rate. 1-year AF symptom recurrence indicates the possibility of potential AF recurrence even though it was not diagnosed at the time of electrocardiography examination at each follow-up time point. Yoshinori et al. found that patients who complained of AF- symptoms underwent more RFCA procedure than those with under-recognized symptoms (32% vs. 13%) [20]. Symptoms are subjective experiences that include physical and psychological changes, but since atrial fibrillation treatment strategies are selected based on the existence and severity of symptoms [21], symptoms that affect quality of life after the procedure are also important factors in determining future treatment directions. Our results may guide that the deep neuromuscular blockade could be a better choice for improvement of symptoms and quality of life after RFCA under general anesthesia.

The German RFCA data revealed that the type of AF, female sex, in-hospital relapse of AF, and decreased renal function are predictors of disease recurrence after RFCA [22]. While these predictors are unchangeable, anesthetic factors such as neuromuscular blockade depth are controllable. Therefore, our findings are valuable for anesthetic management and improving the outcomes of RFCA. The deep neuromuscular blockade may be associated with lower recurrence of AF and symptoms during the 1-year follow-up.

## Limitations

This prospective randomized study had several limitations. First, the follow-up period of patients in our study was 12-months, and long term outcomes could not be identified. However, it was possible to minimize the bias between the two groups, and a significant difference in outcome was confirmed despite the short term follow-up period. Second, although we employed a robust randomization process with sequential numbers concealed in opaque envelopes, the fact that the anesthesiologists who opened the envelopes were not blinded may have introduced allocation bias. However, this was an essential step to ensure the accurate adjustment of the muscle relaxant dosage, and all other data were recorded and managed by personnel not involved in the study. Third, we did not collect data on continuous catheter contact force, which not only affects the AF recurrence rate, but also affects procedural parameters such as fluoroscopy time and ablation time [5,23-25]. In the future, it is also necessary to study the difference in contact force according to the degree of neuromuscular block and the outcomes accordingly.

## Conclusion

Compared to moderate neuromuscular blockade, deep neuromuscular blockade while performing RFCA under general anesthesia reduced 12-month AF recurrence rate. Deep neuromuscular blockade under general anesthesia is thought to increase the success rate by providing a stable surgical environment during the RFCA procedure. However, further large-scale and long-term trials are required to support our results.

## Supporting information

S1 File Research protocol. (DOCX)

S1 Checklist CONSORT checklist. (DOC)
