# Supplementary material for: Deep neuromuscular blockade during radiofrequency catheter ablation under general anesthesia reduces the prevalence of atrial fibrillation recurrence when compared to moderate neuromuscular blockade: A randomized controlled trial
Source: PLoS One. 2025 Jan 21;20(1):e0302952. doi: 10.1371/journal.pone.0302952 (PMC11750084; doi:10.1371/journal.pone.0302952)
Supplement: S1 File — (DOCX) [file pone.0302952.s001.docx]

# Research Protocol

#### Project summary

The study aims to compare the procedural conditions and outcomes based on the degree of muscle relaxation in patients undergoing RFCA under general anesthesia. Conducted at Samsung Seoul Hospital, the research involves adult patients aged 19-70 years with ASA class I-IV, undergoing RFCA for atrial fibrillation for the first time. The primary endpoint is to compare the recurrence rates based on muscle relaxation levels using Wilcoxon rank test, Cox proportional hazard model, and Kaplan-Meier method for visualizing 1-year follow-up results. Secondary endpoints include assessing HR, BP, BIS through repeated measures ANOVA, and evaluating surgery duration, self-respiration recovery frequency, and operator satisfaction score. The research hypothesis suggests that the degree of muscle relaxation during RFCA under general anesthesia affects procedural safety and post-procedure outcomes. The study will control the dosage of muscle relaxants to verify the recurrence rate of atrial fibrillation by comparing moderate and deep blocks. Data collection includes patient characteristics, hemodynamic variables, procedural times, and adverse effects, with a follow-up period of one year to assess procedural success and operator satisfaction.

#### General information

**Title:** **Deep neuromuscular blockade during radiofrequency catheter ablation under general anesthesia reduces the prevalence of atrial fibrillation recurrence when compared to moderate neuromuscular blockade: a randomized controlled trial**

****** *The title registered in the clinical trial is named “Comparison between the effects of deep and moderate neuromuscular blockade during RFCA(Radiofrequency catheter ablation) on procedure condition and outcomes: a prospective, randomized, and controlled trial”*

- **Investigator**
- Jin Hee Ahn
- **Principal Investigator**

**Ji Seon Jeong,** M.D., Ph.D.

Tel.: +82-2-3410-2463; Fax: +82-2-3410-2461; E-mail: [jiseon78.jeong@samsung.com](mailto:jiseon78.jeong@samsung.com)

- **Institution**

Department of Anesthesiology and Pain Medicine, Samsung Medical Center, Sungkyunkwan University School of Medicine, Seoul, Korea

#### Rationale & background information

Radiofrequency catheter ablation is primarily performed on patients with A-fib or A-flutter (atrial fibrillation or atrial flutter), and for A-fib ablation, PV (pulmonary vein) isolation is the main procedure. During PV isolation, patients mainly complain of pain, and recently there has been a trend shifting from simple sedation to general anesthesia.1,2 In general anesthesia, the use of muscle relaxants is essential to maintain mechanical ventilation. For endotracheal intubation, a dose of 0.5-1.0 mg per kg is administered, and during the surgery, a maintenance dose of 0.1-0.2 mg per kg is given at one-hour intervals or when spontaneous breathing (as seen on the capnogram) occurs. This dosage is consistent with the standard dosage for general anesthesia. Currently, there are no specific guidelines for muscle relaxant administration in RFCA.

Empirically, in such RFCA procedures, spontaneous breathing frequently occurs during inhalation anesthesia with mechanical ventilation, and the additional administration of rocuronium is more frequent compared to other surgeries. This is because spontaneous breathing often occurs not only during PV isolation but also during radiofrequency ablation of surrounding tissues, particularly around the SVC. The phrenic nerve, which runs near the SVC and IVC, is often stimulated by this electrical activity, leading to frequent spontaneous breathing. Meanwhile, the mismatch between positive pressure from mechanical ventilation and spontaneous breathing can cause the mapping location (the part marked in advance for PV isolation) to shift, requiring multiple re-mappings and extending anesthesia time. Therefore, it is necessary to maintain appropriate anesthesia and muscle relaxation levels through continuous administration of muscle relaxants.

The FDA-approved dosage for continuous infusion of rocuronium is 4-16 mcg/kg/min (see rocuronium pharmacopeia attachment), but the recommended domestic dosage is 5-12 mcg/kg/min under inhalation anesthesia. It is thought that differences in the degree of muscle relaxation could impact post-procedure outcomes and the convenience of the procedure for the operator, which led to the planning of this study. Previous studies analyzing the recurrence rates between general anesthesia and simple sedation found that recurrence rates were lower with general anesthesia.1,2 However, there are no current studies on the recurrence rate of A-fib according to the degree of muscle relaxation during general anesthesia.

A retrospective analysis of 45 patients at our hospital with varying degrees of muscle relaxation during general anesthesia showed a 1-year recurrence rate of 0.04 (1 out of 25 patients) in the moderate group and 0.25 (4 out of 20 patients) in the deep block group. Previous studies have shown that high doses of rocuronium can result in QT prolongation.3 This affects ventricular conduction, which relates to the possibility of ventricular arrhythmia, but it is also necessary to consider its potential effect on atrial conduction, reducing the occurrence of ectopic beats during the procedure. (See references a and b below.) Therefore, this study was planned to clearly control the dosage of muscle relaxants and verify the recurrence rate of A-fib when divided into moderate block and deep block groups according to the degree of muscle relaxation.

#### References (of literature cited in preceding sections)

Ref a) Effects of High-Dose Rocuronium on the QTc Interval During Anaesthesia Induction in Patients Undergoing Coronary Artery Bypass Graft Surgery

Ref b) Long QT syndrome and anaesthesia[P.D.Booker](https://www.sciencedirect.com/science/article/pii/S0007091217368253?via%3Dihub#!)[S.D.Whyte](https://www.sciencedirect.com/science/article/pii/S0007091217368253?via%3Dihub#!)[E.J.Ladusans](https://www.sciencedirect.com/science/article/pii/S0007091217368253?via%3Dihub#!)

#### Study goals and objectives

#### Safety and Post-Procedure Outcomes of Radiofrequency Catheter Ablation under General Anesthesia Based on the Degree of Muscle Relaxation during the Procedure

#### Study Design

- **Design** : Prospective and Randomized controlled Trial
- **Research population** : Adult
- **Inclusion criteria**
  - 1) Adult patients aged 19-70 years with ASA class I-IV
  - 2) Patients diagnosed with atrial fibrillation undergoing RFCA under general anesthesia for the first time
- **Exclusion criteria**
- Pediatric patients (under 19 years old)
- Patients previously diagnosed with atrial fibrillation who have undergone RFCA
- Patients with a high risk of pulmonary aspiration
- Patients with unstable vital signs
- Patients with liver or kidney disease
- Patients with metabolic disorders
- Emergency surgery
- Pregnant or breastfeeding women
- **Expected duration of study** : 1 year after IRB approval

#### Methodology

- After the patient is admitted, full monitoring (EKG, NIBP, SpO2) is conducted, followed by O2 preoxygenation.
- Administer propofol 2 mg/kg as a sedative drug and confirm the loss of consciousness. After TOF watch calibration, administer the muscle relaxant rocuronium 0.4 mg/kg.
- After 1 minute and 30 seconds, insert the I-gel (Laryngeal mask) and set mechanical ventilation to 8 ml/kg, ensuring that airway pressure does not exceed 20 mmHg. (If it exceeds 20 mmHg, adjust the position of the I-gel.)
- After induction of anesthesia, monitor the TOF ratio at 5-minute intervals. When TOF is 1 or 2, start continuous infusion of rocuronium. Repeatedly monitor TOF at 5-minute intervals.
- For Group M, start the initial infusion dose at 5 mcg/kg/min and maintain TOF 1-2 under TOF monitoring. If TOF is 0, decrease by 0.5; if TOF increases to 3-4, increase by 0.5. At the end of the procedure, administer sugammadex 2 mg/kg and perform extubation.
- For Group D, start the initial infusion dose at 8 mcg/kg/min and maintain TOF 0 under TOF monitoring. Maintain TOF 0, PTC below 2, and if TOF increases to 1-2, increase by 0.5. At the end of the procedure, administer sugammadex 4 mg/kg and perform extubation.

#### Safety Considerations

#### Moderate degree of adverse events associated with the study procedure

#### Severity of the adverse reaction is evaluated by the following criteria.

#### ① Mild: It can be tolerated easily even though it has subjective or other symptoms.

#### ② Moderate: It is uncomfortable enough to interfere with daily life.

#### ③ Severe: To be able to perform normal daily life

#### The anesthesiologist specialist JHA will perform close monitoring if the change is observed after surgery. If serious adverse events are observed, they should be reported to the IRB and treated in accordance with in-hospital treatment standards.

#### During the study period, the researcher (anesthesiologist) examines and measures and performs vital monitoring. <Confidentiality of the subject> The record of the subject's identity will be kept confidential and the identity of the subject will remain confidential even when the results of the clinical trial are published. The subject's charts and case record information are kept confidential and kept in a confidential facility and in accordance with its management standards. <Method of coding data> In all documents related to the clinical trial, such as case records, record and distinguish the patient's identification code (usually Case No.) rather than the patient's name.

#### Data Management and Statistical Analysis

- **Data** : All patient information is coded and documented.
- **Sample size calculations** : Our unpublished retrospective data showed that in the Moderate group, the 1-year recurrence rate was 0.04, with 1 out of 25 patients experiencing recurrence. In the Deep block group, the recurrence rate was 0.25, with 4 out of 20 patients experiencing recurrence. The analysis was performed using the Z test with an alpha error of 0.05 and a power of 0.8. Therefore, each group will consist of 43 patients, and with an estimated dropout rate of 10%, each group will have 48 patients, making a total of 96 patients.
- **0.1z tests -** Proportions: Difference between two independent proportions
- **Analysis:** A priori: Compute required sample size
- **Input:** Tail(s) = Two
- Proportion p2 = 0.04
- Proportion p1 = 0.25
- α err prob = 0.05
- Power (1-β err prob) = 0.80
- Allocation ratio N2/N1 = 1
- **Output:** Critical z = -1.9599640
- Sample size group 1 = 43
- Sample size group 2 = 43
- Total sample size = 86
- Actual power = 0.8006434
- **Statical analysis**

#### Primary Endpoint

#### Comparison of recurrence rates based on the degree of muscle relaxation

#### Evaluation methods:

#### ① Using the Wilcoxon rank test to determine the median survival time of both groups and compare the recurrence rates between the two groups to see if there is a significant difference.

#### ② Using the Cox proportional hazard model to verify whether the continuous dose of rocuronium is a variable affecting survival time and recurrence rate.

#### ③ Follow-up results for 1 year after the procedure will be visualized by drawing a survival curve using the Kaplan-Meier method.

#### Secondary Endpoint

#### Repeated measures ANOVA: HR, BP, BIS

#### Student's t-test: surgery duration, number of self-respiration recoveries, operator satisfaction score

#### Quality Assurance

#### 1) Preservation of documents related to clinical trials: The institution conducting clinical trials shall preserve various data (including electronic documents) related to the conduct of clinical trials, including clinical trial plans and records on the management of clinical trial procedures, for a period of 10 years from the end of the study .

#### 2) Confidentiality: Confidentiality of data and test subject records: All test subject identification information must be kept confidential, and the case record will include the test subject number and the test subject initials. The subject will be able to view the subject's medical records for the purpose of identifying the information collected by the IRB and the Food and Drug Administration and will inform that the information will be handled in a strictly confidential manner.

#### 3) Clinical Trial Management Standards (KGCP, ICH E6): The procedures set out in this plan are based on the Clinical Trial Management Standards (KGCP: ICH E6) for testing, ) And the fundamental spirit of the Helsinki Declaration.

#### 4) Clinical trial review committee (IRB): Before starting the trial, the examiners should submit a copy of the clinical trial plan, the consent form, the data and procedures related to the recruitment of test subjects, It must be approved. The IRB's decision on the conduct of the test shall be communicated to the tester prior to commencement of the test. The person responsible for the clinical trial will report to the IRB on the progress of the trial and any serious adverse events, life-threatening problems or missions, and notify the IRB at the end of the other tests.

#### 5) Test subject's consent: The examiner should explain to the test subject (and / or his / her representative) participating in the clinical trial so that they can easily understand the nature of the test, the expected results, etc., , The test subject's written consent shall be signed and dated by the examiner and the subject and / or the agent. The original signed agreement must be kept by the tester and a copy must be given to the subject or the subject's representative. The examiner should not carry out any tests for the purpose of clinical studies before obtaining consent from the subject.

#### 6) Surveillance Survey: The purpose of surveillance surveillance surveillance surveys is to ensure that all research activities and documents related to the research are carried out in accordance with the protocol, GCP, ICH guidelines and other regulatory requirements. And to examine them systematically and independently. The relevant authorities and the Food and Drug Administration may request that the relevant documents, case records, and other test documentation be viewed for inspection or surveillance, and the tester shall permit and cooperate with this process at all times. The researcher should immediately contact the relevant department of the regulatory agency if the investigation is scheduled.

#### Expected Outcomes of the Study

There are no guidelines yet to address head and neck postures for proper tracheal intubation in children. Therefore, the appropriate level of shoulder elevation and traction to improve the laryngeal view for each individual will be different, and HA-ECTS can be used to improve the laryngeal view more easily. Therefore, this study will provide useful clinical information for pediatric intubation.

#### Dissemination of Results and Publication Policy

#### Corresponding author: Ji Seon Jeong, M.D., Ph.D

#### Duration of the Project

The study period is one year after the clinical trial approval. However, when the study is completed before that time, the time point is the end point of the study.

#### Project Management

Jin Hee Ahn : Patient collection, Manuscript writing

Ji Seon Jeong : Study design, Manuscript writing, critical comments

#### Ethics

The investigator explained to the patient one day before the anesthesia all protocols for this study for more than 30 minutes, and the informed consent was obtained after the patient understood it all

#### Informed Consent Forms

*Attached as additional files

#### Budget

#### This research received no specific grant from any funding agency in the public, commercial, or not-for-profit sectors.

#### Other support for the Project

None.

#### Collaboration with other scientists or research institutions

None.

#### Curriculum Vitae of investigators

**Attached as additional files

#### Other research activities of the investigators

The Principal investigator (JSJ) contributed over 50% to study design and overall research participation in this study.

#### Financing and Insurance

#### Financing and insurance is based on the Samsung Medical Centre compensation standard.
